# Author's response—III

**Published:** 2006

**Authors:** Paul Hoff

**Affiliations:** * Professor of Psychiatry, Deputy Medical Director Department of General and Social Psychiatry, University of Zurich, Lenggstr. 31, P.O. Box 1931, CH 8032 Zurich, Switzerland; e-mail: paul.hoff@puk.zh.ch

Sir,

In response to Dr Basu's and Dr Chatterjee's commentaries I would like to briefly address two points:

1. Both colleagues are perfectly right when stating that it is *not* justified to simply identify Foucault's writings with ‘antipsychiatry’—and such an identification is not what I intended. However, Foucault's critical discussion of the close relationship between psychiatric practice and the public and political opinion about normality, madness and the resulting methods to define the latter and reinforce psychiatric procedures, was, of course, attractive to ‘antipsychiatric’ authors as a theoretical framework—even if they had not in any case reflected about Foucault's writings extensively.
2. More important, in my view, is the second point, which deals with the central ideas of enlightenment: On the one hand, it is true that a predominantly rationalistic reading of the ideas of enlightenment will create a flawed perspective on human subjectivity and behaviour by grossly under-estimating affective and volitional aspects. On the other hand, enlightenment itself was by no means homogeneous in that respect. Especially I. Kant's and J.G. Fichte's writings—mostly labelled as ‘transcendental idealism’—explicitly acknowledge the essential impact of volition, affect and interpersonal relationship not only on practical aspects within societies, but also on any possible theoretical concept of man. So, in summary, I agree with Dr Basu that a narrow rationalistic interpretation of enlighten-ment is not helpful, especially not for psychiatric issues. But the central idea of enlightenment, *personal autonomy and responsibility,* which is *not* restricted to rationalistic arguments, is of great value for psychiatry when it comes to difficult and controversial issues like the concept of illness or the degree of autonomy in the presence of a mental disorder. This debate should be strongly encouraged in present day psychiatry—be it of eastern or western origin.

